# Panel discussion: The challenges of translating evidence into policy and practice for maternal and newborn health in Ethiopia, Nigeria and India

**DOI:** 10.1186/1472-6963-14-S2-O7

**Published:** 2014-07-07

**Authors:** Meenakshi Gautham, Della Berhanu, Nasir Umar, Amit Ghosh, Noah Elias, Neil Spicer, Agnes Becker, Joanna Schellenberg

**Affiliations:** 1 Faculty of Infectious & Tropical Disease, London School of Hygiene & Tropical Medicine, London WC1E 7HT, UK; 2 Mission Director, National Rural Health Mission, State Project Management Unit, 19-A Vidhan Sabha Marg, Lucknow -226001, India; 3 Director, Policy & Planning Directorate, Federal Ministry of Health, Addis Ababa, Ethiopia

## Background

Maternal and newborn deaths are unacceptably common in Ethiopia, North-Eastern Nigeria, and the state of Uttar Pradesh in India. Governments are working to strengthen health systems to improve maternal and newborn health but need access to accurate evidence on which to base decisions. This panel session will include both policy-makers and researchers and include examples of how they can work together to translate evidence into policy and practice. Three brief examples are given below. The discussion will draw from these and others, building on a framework from our recent qualitative study of what helps and hinders the scale-up of health innovations in within the health systems in these settings. The session will be interactive, with active participation of audience members.

## Involving government in Ethiopia

A close partnership between researchers and government can facilitate the use of evidence in decision-making. In Ethiopia, the Federal Ministry of Health lacks skilled health professionals who could help to synthesize evidence for policy-making. Moreover, at all levels of the health systems there is little culture or tradition of trusting or using evidence. For example, a variety of prevalence rates have been reported for mother to child transmission of HIV in Ethiopia, and it has been challenging for policy makers to decide which evidence to use to inform policies to strengthen health systems.

## Involving government in Uttar Pradesh, India

In Uttar Pradesh a similar barrier was overcome by researchers aligning with government health policies and systems and seeking the Mission Director’s involvement in research work. This led to local research evidence contributing to a directive to all health facilities promoting delayed bathing of the newborn, a practice which is proven to save lives. The Mission Director would like further evidence on how to address the barriers that are impeding acceleration of the decline in infant mortality rates.

## Community acceptance and engagement with traditional and religious leaders

In the context of weak health systems it can be particularly important to engage with community leaders. For example, in North-East Nigeria, the Society for Family Health work with traditional birth attendants and with volunteers from a local Muslim women’s association to strengthen health systems as a way to improve life-saving childbirth care practices.

## Conclusions

This session will give the audience an awareness of the challenges facing both researchers and policy makers in promoting evidence-based practice and provide them with clear examples of *how* to translate evidence into health systems policies in three low-resource settings.

